# Self-esteem mediates the relationship perceived stigma with self-efficacy for diabetes management in individuals with type 2 diabetes mellitus

**DOI:** 10.15537/smj.2022.43.10.20220344

**Published:** 2022-10

**Authors:** Ayfer Ozturk, Semih Akin, Necla Kundakci

**Affiliations:** *From the Department of Nursing (Ozturk), Faculty of Health Sciences; from the Vocational School of Health Services Department of Health Care Services Aged Care Program (Kundakci), Vocational School of Health Services, Bartin University, Bartin, and from the Department of Nursing (Akin), Nursing Faculty, University of Health Sciences, Istanbul, Turkey.*

**Keywords:** type 2 diabetes mellitus, stigmatization, self esteem, self efficacy

## Abstract

**Objectives:**

To determine the mediating effect of self-esteem in the relationship between the perceived stigmatization of individuals with type 2 diabetes mellitus (T2DM) and their self-efficacy regarding diabetes management.

**Methods:**

The study was carried out with 162 patients with T2DM who visited the Internal Medicine outpatient clinic, Bartin Public Hospital, Bartin, Turkey, between December 2020 and May 2021. A descriptive information form, diabetes management self-efficacy scale, Rosenberg self-esteem scale, and type-2 diabetes stigma assessment scale were used in data collection.

**Results:**

As a result of regression analyses, it was determined that the variables of stigmatization (ß= -0.294) and self-esteem (ß=0.875) had a significant predictive effect on self-efficacy of patients with T2DM, and that as self-esteem was added to the model, the effect of stigmatization on self-efficacy (ß= -0.294) decreased (ß= -0.230, *p*<0.05). According to these findings and the results of the Sobel test, it was determined that self-esteem had a partial mediator role (z= -3.347; *p*< 0.05).

**Conclusion:**

Minimizing the perceived stigmatization can improve patients’ diabetes management self-efficacy. With patient training programs and individualized nursing care plans prepared by psychiatric nurses to provide psychological support patients and through their interventions that increase self-esteem, self-stigmatization can be reduced.

**D**iabetes mellitus (DM) is a global health problem that threatens the entire world whose prevalence is increasing fast. According to the data of the International Diabetes Federation (IDF), there were 537 million people with diabetes in the world in 2021. It is estimated that this number will reach approximately 643 million in 2030 and 783 million in 2045.^1^ Moreover, according to IDF, approximately 90% of diabetes cases consist of type-2 diabetes mellitus (T2DM), and one in every 11 people in the world have diabetes.^1^

Diabetes is not only a disease that progresses with physical symptoms but also a disease that has psychiatric and psychosocial aspects.^2^ Such individuals may also experience psychological problems due to pressure and stigmatization by people around them.^3^

Stigma is founded on cognitive, emotional, and behavioral reactions to individuals with some diseases due to the cognitive schemas and prejudices of society against some patient groups. Stigma may be at least as dangerous as the disease itself. The stigmatized person is attributed a characteristic that is not based on facts and is infamizing.^4^ This social stigma, in time, leads to the person’s development of stigmatizing attitudes towards self.^5^ Self-stigma affects the mental health of the individual and their feelings on healthy living negatively.^5^ If the fact that genetic and environmental factors playing a role in the development of T2DM is neglected, perceptions that this disease is only related to the person’s lifestyle emerge. In this case, diabetes is perceived as a situation that is under the control of the diagnosed person, and thus, patients may think that they have caused their diabetes diagnosis by themselves.^6^

Studies carried out with people with diabetes have revealed that patients experience serious levels of stigma regarding the disease.^3,7-10^ In their study carried out with 12,000 Americans with type 1-2 diabetes, Liu et al^7^ reported high levels of perceived stigma in the people with diabetes. In another qualitative study carried out by Browne et al,^9^ people with T2DM shared their stigmatization experiences and described DM as a disease involving shame and blame. Such prejudiced and stigmatizing attitudes regarding the lifestyle that causes the disease affect disease-related self-management and the disease process negatively.^9^

Diabetes management requires treatment compliance and the person to make some behavioral changes in their daily life. In addition, studies have shown that stigma perceived by people with diabetes affects their DM management negatively.^5,11-14^ Kato et al^5^ reported that internalized stigma had a negative effect on the self-management of people with T2DM. In another study by Kato et al^13^ a negative relationship was found between the self-stigma levels and self-care behaviors of people with T2DM. In the study carried out by Lin et al^14^ with 115 people with T2DM, the authors reported that the self-stigma perceptions of the patients affected their DM-related self-care behaviors negatively.

Self-esteem, which is one of the main elements of the concept of self, was defined as the person’s value, embracement, trust, and respect for themselves.^15^ It was reported that individuals with high levels of self-esteem had better compliance with self-care activities.^16^ A study showed that the stigma perceived by people with T2DM led to a reduction in their self-esteem and their self-efficacy in DM management.^17^

It is known that individuals with high self-esteem show better compliance with DM-related self-care activities. In addition to this, the perception of stigma in people with diabetes affects their self-management of DM negatively. It has been reported that there is a negative relationship between stigma and self-esteem, where self-esteem decreases as stigma increases. From this perspective, it is considered that the variable of self-esteem plays a role as a mediator variable in the relationship between the perceived stigma and DM-related self-efficacy people with diabetes. In other words, the effect of the stigma perceived by people with diabetes on their self-efficacy may change when self-esteem is included in this relationship. There are limited number of studies on this subject in the literature.

From this perspective, in this study, primarily it was aimed to investigate the predictive effects of self-esteem and stigma in individuals with T2DM on their perceived self-efficacy in the management of DM. The second purpose of this study was to examine the mediating effects of self-esteem in the relationship between the stigma perceived by people with T2DM and their perceived self-efficacy related to DM management.

## Methods

This study was carried out with an analytic cross-sectional design. The study was carried out with 162 patients with T2DM who visited the Internal Medicine outpatient clinic, Bartin Public Hospital, Bartin, Turkey, between December 2020 and May 2021. The STROBE guidelines for cross-sectional studies were followed. The population of the study consisted of all patients diagnosed with T2DM who attended examinations within a year in the Internal Medicine outpatient clinics, Bartin Public Hospital, Bartin, Turkey. As the total number of cases within a year was not exactly known, a power analysis was carried out using the G^*^Power, version 3.1.7 software. The effect size was obtained as a moderate effect size (f2=0.15) according to the multiple regression analysis reported by Cohen.^18^ It was calculated that the study should include at least 107 individuals to obtain a power of 95% with an effect size of 0.15 and a significance level of 5%. The study was completed with 162 patients. At the end of the study, the effect size was 0.5 (*p*=0.05).

Patients were selected with the convenience sampling method based on whether they met the following inclusion criteria: I) being diagnosed with T2DM; II) taking medication (such as oral antidiabetic and insulin therapy); and III) voluntarily agreeing to take part in the study. We excluded patients with a diagnosis of type 1 diabetes, gestational diabetes mellitus, younger than 18, having psychiatric illness, and not taking medication.

The data were collected through face-to-face interviews with diagnosed T2DM patients after they had been informed on the research process. Before the surveys were distributed by the researcher, the interviewee was informed regarding the purpose of the study, the inclusion/exclusion criteria for sampling, the research process, and the content of the surveys. All patients provided written informed consent for their participation before study entry.

Ethical approval to carry out the study was obtained from the Bartin University Ethics Committee (date: 12.07.2019, approval number: 2019/156), and permissions were obtained from the Provincial Health Directorate (date: 03.09.2019, No.: 78239813-799). Before carrying out this study, permission to use the scales was obtained from their original developers by e-mail. The patients were informed regarding the purpose of the study, its content and that the data would only be used for scientific purposes. Identifying information was not requested from the patients. The study was carried out according the principles of Helsinki Declaration.

### Measures

Descriptive Information Form included questions to collect information on the sociodemographic characteristics (such as age, gender, marital status, and educational level) and diabetes management-related characteristics (duration of diabetes, history of diabetes in first- and second-degree relatives, status of having received education on diabetes, regular health follow-up status, exercise status, and diabetic diet status) of the patients.

Rosenberg Self-Esteem Scale (RSES) which was developed by Rosenberg,^19^ consists of a total of 63 questions under 12 categories. The first 10 items of the Turkish form of the scale adapted by Çuhadaroğlu,^20^ measured the self-esteem dimension. In this study, to determine the self-esteem levels of the patients, these 10 items were used. This part of the scale containing 5 positive and 5 negative statements has a 4-point Likert-type scoring system. The scores of the form vary between 10-40 after the inversely scored items are converted, and higher scores represent higher levels of self-esteem. As self-esteem is assumed to be a one-dimensional concept the total score was used in this study. In the reliability study carried out by Rosenberg,^19^ the test-retest reliability coefficients of the dimensions of RSES were found to be in the range of 0.82-0.88, while the Cronbach’s alpha coefficients of the dimensions were in the range of 0.77-0.88. In this study, the Cronbach’s alpha internal consistency coefficient for the self-esteem dimension that was used was identified as 0.78.

Type 2 Stigma Assessment Scale which was developed by Browne et al,^21^ is a self-report scale that assesses perceived and experienced stigma in adults with T2DM. The scale that was tested for validity and reliability by Can Gür et al^6^ consists of 19 items and 3 dimensions. These dimensions are: I) different behaviors (1^st^-6^th^ items); II) blame and judgment (7^th^-13^th^ items); and III) self-stigmatization (14^th^-19^th^ items). Each item is scored as a 5-point Likert-type scale in the form of: 1=“absolutely disagree”; 2=“disagree”; 3=“undecided”; 4=“agree”; and 5=“absolutely agree”. The range of the possible total scores of the scale is 19-95, and higher scores in each dimension indicate more severe stigmatization of the person. The Cronbach’s alpha coefficient of the original version of the scale was reported as 0.95 while it was calculated as 0.91 in this study.

Diabetes Management Self-Efficacy Scale was developed by Bijl et al^22^ to determine the perception of people with diabetes regarding their capacity to carry out self-care activities in their management of T2DM, and it was tested for validity and reliability by Usta Yeşilbakan.^23^ It consists of 20 items and is a 5-point Likert-type scale (1=strongly disagree; 2=disagree; 3=somewhat agree; 4=agree; and 5=strongly agree). The minimum and maximum scores of the scale are 20 and 100. Based on the general average score obtained from the item average scores of all subscales, those who have scores under the general average score were considered to have low self-efficacy, while those with scores over the general average score were considered to have high self-efficacy. The Cronbach’s alpha coefficient of the scale was reported as 0.89.^23^ In this study, the Cronbach’s alpha coefficient was calculated as 0.96 for the scale.

### Statistical analysis

The data collected in this study were analyzed using the Statistical Package for the Social Sciences, version 22.0 (IBM Corp., Armonk, NY, USA). Descriptive statistics were presented as frequencies and percentages. Skewness and kurtosis values were examined to determine whether the data were normally distributed. In the relevant literature, data were accepted to be normally distributed if the skewness and kurtosis values are in the range of -1.5 - +1.5 or -2.0 - +2.0. In this study, the skewness and kurtosis values were found to be within the specified reference ranges. Additionally normality was tested using the Komogorov-Smirnov (KS=0.007). As the data found to be normally distributed, parametric tests were used in the analyses. The continuous variables of the study were subjected to Pearson’s correlation, linear regression, and hierarchical regression analyses. To test the significance of the mediating effect, we used the Baron and Kenny method^24^ and the Sobel test. The level of statistical significance was set at *p*<0.05 for all analyses. In the Sobel, the full or partial mediation status of a variable was determined by measuring the reduction in the rate of the total variance explained by the independent variable.^25^ The relative contributions of self-esteem and stigma in predicting diabetes management-related self-efficacy were tested using multiple regression analysis. The variance inflation factor (VIF) values that show the degree of multicollinearity must be smaller than 10, and the tolerance value must be greater than 0.1. The Durbin-Watson value, referring to auto-correlation, must be in the range of 1.5-2.5. No auto-correlation was identified between the independent variables (1.5<DW>2.5). Moreover, the tolerance and VIF values showed the absence of a multicollinearity problem (T>0.1; VIF<10). Data were analysed using a 3-step hierarchical regression analysis and the Sobel test.

## Results

It was determined that 36.4% of the patients were at the ages of 41-50, and 57.4% were male. While 83.3% were married, 49.4% were primary school graduates. Almost half (46.9%) had been with diabetes for 1-5 years, and 40.7% had a history of DM in their first-degree relatives.

The vast majority (82.1%) stated that they had previously received diabetes education. More than half (54.9%) of those who had received diabetes education reported that they did not find the education they had received adequate.

In the sample, 83.3% were using oral antidiabetic medication for treating their DM. While 86.4% said they used their medication regularly, 52.5% stated that they did not follow a diabetic diet, and 52.5% reported that they did not regularly exercise (Table 1).

**Table 1:** - Sociodemographic and disease-related characteristics of the participants (N=162).

Variables	n (%)
***Age, mean±SD (minimum-maximum)***	49.59±9.30 (28-65) years
40 years old or younger	32 (19.8)
41-50 years old	59 (36.4)
51-60 years old	50 (30.9)
Older than 60 years old	21 (13.0)
***Gender***	
Female	69 (42.6)
Male	93 (57.4)
***Marital status***	
Married	135 (83.3)
Single	27 (16.7)
***Education level***	
Primary school	80 (49.4)
Secondary school	20 (12.3)
High school	41 (25.3)
University or higher	21 (13.0)
***Duration of diabetes***	
Shorter than one year	62 (38.3)
1-5 years	76 (46.9)
Longer than 5 years	24 (14.8)
***Family history of diabetes***	
Yes, in first-degree relatives	66 (40.7)
Yes, in second-degree relatives	62 (38.3)
No	34 (21.0)
***Has received diabetes education?***	
Yes	133 (82.1)
No	29 (17.9)
***Has received adequate education about diabetes? (n=133)***	
Yes	60 (45.1)
No	73 (54.9)
***Attends regular health follow-ups?***	
Yes	136 (84.0)
No	26 (16.0)
***Treatment method***	
Only oral antidiabetic medication	135 (83.3)
Only insulin	18 (11.1)
Oral antidiabetics and insulin	9 (5.6)
***Uses medication regularly?***	
Yes	140 (86.4)
No	22 (13.6)
***Regular exercise habit?***	
Yes	27 (16.7)
Sometimes	50 (30.9)
No	85 (52.5)
***Follows a diabetic diet?***	
Yes	77 (47.5)
No	85 (52.5)

Values are presented as a number and precentage (%). SD: standard deviation

Correlations between the perceived stigma, self-esteem, and diabetes management self-efficacy levels of the patients were analyzed. According to the analysis results, there was a negative correlation between self-esteem and perceived stigma (r= -0.29, *p*<0.05). There was also a negative correlation between self-efficacy and perceived stigma (r= -0.25, *p*<0.05). Additionally, a positive correlation was identified between self-efficacy and self-esteem (r=0.25, *p*<0.05; Table 2).

**Table 2:** - Correlation analysis between stigma, self-esteem, and diabetes management self-efficacy.

Scales	Stigma total scores	Self-esteem total scores	Diabetes management self-efficacy total scores
Stigma total scores	r=1.000		
	*p*=0.000		
Self-esteem scores	r= -0.29	r=1.000	
	*p*=0.000	*p*=0.000	
Diabetes management self-efficacy total scores	r= -0.25	r=0.25**	r=1.000
	*p*=0.001	*p*=0.001	*p*=0.000

In Model 1, regression analysis was carried out to determine the effect of self-esteem on self-efficacy in diabetes management, and the results are given in Table 3. According to the ANOVA results that tested the validity and significance in the regression analysis, the F value for self-esteem was calculated as 10.464 and the significance value was calculated as *p*=0.001 at the 5% significance level. The R^2^ value of the independent variable, which is the level of explanation of the dependent variable, was calculated as 0.056. According to this result, 5.6% of the change in self-efficacy is explained by self-esteem. It was found that self esteem had a positive predictive effect on diabetes management self-efficacy (ß=0.875). A model study to determine the effect of self-esteem on self-efficacy in diabetes is significant as a whole (R^2^=0.056; F=10.464).

**Table 3:** - Correlation analysis between stigma, self-esteem, and diabetes management self-efficacy.

Dependent variables	Independent variables	ß	t	*P*-values	F	Model (*p*-values)	Adjusted R^2^	R^2^ change	ß (95% CI)
Diabetes management self-efficacy (Model 1)	Constant	35.084	4.752	0.000	10.464	0.001	0.056		0.875 (0.342-1.408)
	Self-esteem	0.875	3.235	0.001					
Self-esteem (Model 2)	Constant	32.792	19.963	0.000	14.237	0.000	0.057		-0.095 (-0.145 - -0.045)
	Stigma	-0.095	-3.773	0.000					
Diabetes management self-efficacy (Model 3)	Constant	77.047	13.152	0.000	10.659	0.001	0.057	0.062	-0.294 (-0.472 - -0.116)
	Stigma	-0.294	-3.265	0.001					
Diabetes management self-efficacy (Model 4)	Constant	54.822	5.086	0.000	8.473	0.000	0.085		
	Stigma	-0.230	-2.479	0.014				0.062*	-0.230 (-0.413 - -0.047)
	Self-Esteem	0.678	2.441	0.016				0.034**	0.678 (0.129-1.226)

^*^ Predictors: (constant), stigma.

^**^ Predictors: (constant), stigma, and self-esteem. ß: beta regression coefficient, CI: confidence Interval, F: anavo test, R2: adjusted R square

In Model 2, regression analysis was carried out to determine the effect of stigma on self-esteem, and the results are given in Table 3. According to the ANOVA results that tested the validity and significance in the regression analysis, the F value for stigmatization was calculated as 14.237 and the significance value was calculated as *p*=0.000 at the 5% significance level. The R^2^ value of the independent variable was calculated as 0.057. According to this result, 5.7% of the change in self-esteem is explained by stigma. It was found that perceived stigma had a negative predictive effect on self-esteem (ß= -0.095). A model study to determine the effect of perceived stigma on self-esteem is significant as a whole (R^2^=0.057; F=14.237).

In Model 3, regression analysis was carried out to determine the effect of percived stigma on self-efficacy in diabetes, and the results are given in Table 3. According to the ANOVA results that tested the validity and significance in the regression analysis, the F value for stigmatization was calculated as 10.659 and the significance value was calculated as *p*=0.001 at the 5% significance level. The R^2^ value of the independent variable was calculated as 0.057. According to this result, 5.7% of the change in self-efficacy is explained by perceived stigma. It was found that perceived stigma had a negative predictive effect on self-efficacy in diabetes (ß= -0.294). A model study to determine the effect of perceived stigma on self-efficacy in diabetes is significant as a whole (R^2^=0.057; F=10.659).

The R^2^ value was calculated as 0.085 for Model 4, in which the stigma variable and the self-esteem variable were included in the model. According to this result, 8.5% of the change in self-efficacy in diabetes is explained by stigma and self-esteem (R^2^=0.085; F=8.473; Table 3).

In the process of adding variables to the regression model, the change caused by the self-esteem variable in R^2^ was calculated using the Stepwise method. While it was calculated as R^2^ change =0.62; F=10.659 when there was only stigma variable in the model, it was calculated as R^2^ change=0.034; F=5.957 when self-esteem was added to the model. This change shows that when the self-esteem variable is included in the model, the explanatory power of the model changes by 3.4% (Table 3). Additionally, the negative predictive effect of stigma on self-efficacy decreases when self-esteem intervenes (ß= -0.230) According to these findings and the results of the Sobel test, it was determined that self-esteem was a partial mediator (partial moderator) (Z= -3.347; *p*<0.05). In other words, stigma affects self-efficacy in diabetes management both directly and through self-esteem.

## Discussion

This study was carried out to investigate the mediating role of self-esteem in the relationship between the perceived stigma levels of people with T2DM and their self-efficacy regarding diabetes management. In this study, it was determined that as the self-esteem levels of the people with T2DM increased, their diabetes management self-efficacy levels also increased, thus showing that self-esteem predicted self-efficacy in a positive direction (ß=0.875; *p*<0.01). This result was consistent with similar study findings.^26,27^

Mikaeili et al^26^ determined that people with diabetes with high self-esteem levels had higher levels of self-efficacy related to diabetes self-management. Kenowitz et al^27^ also found that people with diabetes with high self-esteem had better compliance with their insulin treatments and exercise schedules. Based on these results, it may be argued that self-esteem makes the adaptation of people with diabetes to self-care activities easier and increases their self-efficacy levels. To increase the self-esteem of patients, healthcare professionals and particularly health and diabetic educators should help the recognition and expression of emotions by having effective communication with them based on empathy, respect, confidence, and care. It may be helpful to focus on past achievements of the person with diabetes and to use the support of family members.

Another result of this study was that perceived stigma had a negative predictive effect on diabetes management self-efficacy (ß= -0.294). Accordingly, the stigma perceived by people with diabetes affects their self-efficacy in the management of DM negatively, and as the level of stigma perceived by patients increases, this leads their self-efficacy regarding their disease to decrease. This result supported the results of similar studies in the literature.^5,11-14,22,28,29^ Kato et al^13^ reported a strong negative predictive effect of self-stigma on the self-care behaviors of people with T2DM. Brazeau et al^11^ stated that in diabetic young people, stigmatization was associated with lower self-efficacy levels, higher A1c levels, severe hypoglycemia, and reduced feelings of wellbeing. A study carried out on T2DM patients revealed that stigma was a significant predictor of the negative perception of insulin treatment.^30^ In individuals with chronic diseases like DM, self-stigma may affect their diabetes self-management negatively by leading these individuals to evade treatment or reducing their treatment adherence. Additionally, it may prevent these individuals from acting in favor of their care by themselves. Increasing the self-care behaviors of a person with diabetes by itself is not sufficient. These individuals need to develop positive attitudes towards the disease and get the help that will reduce their self-stigmatization. Recently published guidelines for the treatment of diabetes emphasize the importance of accounting for the psychological statuses of patients while managing their diabetes, especially their potential to stigmatize themselves.^31^ While they are providing education for people with diabetes, healthcare professionals and particularly health and diabetic educators should keep stigma in their minds and plan the appropriate precautions. Healthcare professionals for education for people with diabetes should focus on not only the physical but also the psychological wellbeing of patients and encourage the diabetic individual, their friends and family members to ask questions on stigma and share their feelings. In clinical practice, there is a need for routine assessment and interventions regarding self-stigma in person with diabetes. In both clinical and social settings, regular health education for increasing psychological wellbeing through reducing self-stigma is recommended.

Perceived stigma in people with diabetes leads their self-efficacy on their disease to decrease and their self-esteem to decline. In a qualitative study carried out by in-depth interviews with diabetes people, Seo et al^32^ reported that people with diabetes had lower self-esteem than the healthy individuals and negative attitudes regarding themselves. Another study showed the negative predictive effect of stigma on self-esteem in people with diabetes.^17^ In the current study, a negative significant relationship was identified between self-esteem and perceived stigma. Moreover, as a result a regression analysis was carried out to determine the effect of percived stigma on self-efficacy, it was found that perceived stigma had a negative predictive effect on self-esteem.

One of the issues of curiosity in this study was whether or not self-esteem played a mediating role in the relationship between the perceived stigma and diabetes management self-efficacy levels of people with T2DM. While research on this topic is limited, existing studies have reported that stigma reduces the diabetes management self-efficacy levels of people with diabetes not only directly but also by lowering their self-esteem.^17,33,34^ A previous study used a model to determine how self-stigma affected the self-care behaviors of people with diabetes regarding diabetes management, where both the direct effect of the variable and its effect mediated by self-esteem were investigated. The authors demonstrated that self-stigma affected the patient’s activation both directly and under the mediation of self-esteem.^17^ In a study carried out with 501 people with T2DM, Pedrero et al^34^ investigated the mediating role of psychosocial variables in the relationship between perceived stigma and self-management behaviors in people with diabetes, and they reported that self-esteem had a mediating role in this relationship. In similarity to the results of other studies, in this study, it was determined that the effect of perceived stigma in the patients on their diabetes management self-efficacy decreased when the variable of self-esteem was added to the model. In other words, when self-esteem was added as the mediator variable, the effect of stigma on self-efficacy was reduced. According to these results, interventions that increase self-esteem and self-efficacy may reduce self-stigmatization in people with T2DM, and thus, increase patient’s activation for self-care. It was emphasized that interventions that aim to improve self-care behaviors among people with T2DM should continue to directly target stigma, in addition to targeting self-esteem and self-efficacy at the same time.^17^

In order to manage the self-care behaviors of patients for optimizing their treatment outcomes, healthcare professionals should firstly assess the self-stigma levels of people with T2DM. These professionals should also try to encourage patients who stigmatize themselves to develop a positive self-image and a positive sense of sensitivity regarding their disease. Previous studies have provided evidence that interventions towards reducing self-stigma in psychiatric patients are effective in developing their skills for coping with self-stigma, improving their readiness to change their problematic behaviors, raising their self-esteem, and in turn, making their treatment compliance easier.^35^ Intervention programs designed for reducing self-stigmatization may also provide similar favorable effects among individuals diagnosed with T2DM, and they may improve treatment compliance by reducing self-stigma levels through patient’s education programs. It is seen that there is a need for more studies focusing on interventional efforts towards eliminating stigma for both patients and healthcare professionals in relation to people with T2DM. Specific and interventional studies that examine the effectiveness of methods focused on reducing self-stigma among people with T2DM and increase self-esteem and self-efficacy should be carried out.

### Study limitations

This study adopted a cross-sectional research design. In the future, a longitudinal study could be used to investigate the long-term influences of self-esteem and perceived stigma on self-efficacy for diabetes management of individuals with T2DM. For all that, the current study provides preliminary evidence for these results worthy of further investigation in prospective and interventional studies. In addition, the mediating role of self-esteem in larger sample groups can be analyzed using the structural equation model and path analysis.

In conclusion, the results of this study supported the evidence in the literature on the interactive relationships among perceived stigma, self-esteem, and diabetes management self-efficacy in individuals diagnosed with T2DM. It was shown that stigma affected the self-efficacy of the people with T2DM who were included in this study not only directly but also by the mediating effect of self-esteem. The study in which these 3 variables considered together is limited. This study also provides new preliminary evidence for the moderator effect of self-esteem on the negative impact to diabetes management self-efficacy of diabetes stigma. Therefore, while efforts need to be made to reduce the occurrence of diabetes stigma in the future, the evidence presented here suggests that interventions to mitigate the effects of existing diabetes stigma may be warranted.

Minimizing the stigma perceived by people with diabetes may improve their diabetes management self-efficacy and motivate them more to participate in diabetes-related self-care behaviors. For the effective management of DM in people with T2DM, it is important to lower their self-stigma perceptions by designing more effective and innovative education programs.

The strategies to be developed by healthcare professionals in general and psychiatric nurses in particular to reduce the self-stigmatization levels of people with diabetes should include encouraging these patients to speak regarding their negative emotions, promoting their positive thinking, strengthening their capacity to cope with diabetes, increasing their self-esteem through empowerment, and referring them to psychological counseling if needed. The provision of psychological support for people with T2DM by psychiatric nurses who will prepare psychoeducation programs and care plans and their interventions that increase the self-esteem of these patients may lower the self-stigma levels of the patients.
